# Machine learning models based on location-radiomics enable the accurate prediction of early neurological function deterioration for acute stroke in elderly patients

**DOI:** 10.3389/fnagi.2025.1582687

**Published:** 2025-04-23

**Authors:** Xiaoming Fu, Chuanyang Zhang, Hongjie Huang, Changcheng Li, Miaomiao Li, XiaoRan Li, Zhijun Gao, Mingyang Peng, Hui Xu, Wenli Zhu

**Affiliations:** ^1^Department of Radiology, The Affiliated Gaochun Hospital of Jiangsu University, Nanjing, China; ^2^Department of Radiology, Nanjing First Hospital, Nanjing Medical University, Nanjing, China

**Keywords:** stroke, early neurological deterioration, magnetic resonance imaging, machine learning, predict

## Abstract

**Background:**

The timely and accurate identification of elderly stroke patients at risk of early neurological deterioration (END) is crucial for guiding clinical management. The present study aimed to create a comprehensive map of lesion location in elderly stroke, and build a machine learning model integrating location features and radiomics to predict END in elderly stroke patients.

**Methods:**

A cohort of 709 elderly stroke patients from two centers patients were enrolled. Three machine learning models [logistic regression (LR), random forest (RF), and support vector machine (SVM)] based on location features, radiomics, and Loc-Rad were constructed to predict END in elderly stroke patients, respectively. The performance of models was evaluated using the receiver operating characteristic curves (ROC) and decision curve analysis (DCA). The SHapley Additive exPlanations (SHAP) was used to interpret and visualize the impact of the model predictors on the risk of END.

**Results:**

The location maps for elderly stroke patients showed the bilateral cerebellum, left basal ganglia, left corona radiata, and right occipital lobe were significantly associated with END (*p* < 0.05). For three ML algorithms, the Loc-Rad models based on location features and radiomics demonstrated better performance than the separate location and radiomics-based models in the training cohort (*p* < 0.05), and the Loc-Rad model constructed with the RF algorithm performed best, with an AUC of 0.883 and accuracy of 0.888, and showed strong prediction performance in the external validation set (AUC of 0.818; accuracy of 0.811). The SHAP plots demonstrated that the most significant contributors to model performance were related to postcentral gyrus left, superior frontal gyrus right, w−HLH_glcm_Correlation, large vessel occlusion and lateral ventricle_body left.

**Conclusion:**

We constructed comprehensive maps of strategic lesion network localizations for predicting END in elderly stroke patients and developed a predictive ML model that incorporates both location and radiomics features. This model could facilitate the rapid and robust prediction of the risk of END, enabling timely interventions and personalized treatment strategies to improve patient outcomes.

## Introduction

Stroke incidence rises with age, with about one-third of acute ischemic strokes occurring in those aged 80 and above. Elderly patients face more complications, higher in-hospital and 3-month mortality rates, and are less likely to return home compared to younger patients (Song et al., 2024). Elderly patients often have comorbidities, reduced physiological reserve, and a higher likelihood of complications, which can exacerbate the impact of even strokes (Bao et al., 2022; Tian et al., 2023). Therefore, predicting early neurological deterioration (END) in elderly stroke patients is particularly critical due to their increased vulnerability to adverse outcomes. Early prediction allows for the implementation of preventive measures, such as optimizing blood pressure control, managing hyperglycemia, and initiating neuroprotective therapies, which may mitigate the risk of deterioration.

Growing evidence indicates that imaging factors as well as clinical factors could be crucial for determining the risk of END in stroke patients (Harada et al., 2019; Zhou et al., 2024). The American Stroke Association has suggested the use of diffusion-weighted imaging (DWI) in the management of stroke patients (Powers et al., 2019). Preliminary region-of-interest (ROI) analysis (infarct volume) and emerging radiomic approaches involving MRI have been well established for stroke diagnosis, prognosis, and therapeutic intervention (Gerschenfeld et al., 2024; Sorby-Adams et al., 2024; Li et al., 2022). However, recent research has shown that there is only a moderate correlation between the lesion volume and the END (Samuels et al., 2023). Lesion location is another factor that should be considered with respect to brain symptoms and outcome prediction. Preliminary pilot studies have shown that incorporating lesion location and size into patient assessments can provide a more accurate estimation of stroke severity than relying solely on volume measurements (Menezes et al., 2007). Recently, a novel approach known as voxel-based lesion-symptom mapping (VLSM) was shown to quantitatively represent the location of brain lesions in a statistical manner (Moon et al., 2022). The individual’s brain image is normalized to a common spatial template with standard coordinates (e.g., Montreal Neurological Institute (MNI) or Talairach), followed by population-based analysis to calculate the frequency of lesion locations in the normalized space and generate a map displaying the anatomic lesion distribution probability. This analysis enables easy statistical inference with respect to brain location, subsequently leading to the independent prediction of the functional outcomes in acute stroke patients.

Machine learning (ML), a branch of artificial intelligence, has been utilized to create predictive models more effectively than conventional approaches by identifying latent patterns in extensive, intricate datasets (Banerjee et al., 2023). Numerous studies have described the use of machine learning methodologies for enhancing prognosis in stroke patients, developing ML-based models that may have a number of advantages when integrated into clinical settings (Heo et al., 2019; Bonkhoff and Grefkes, 2022). In this study, we first explored the location on DWI associated with END using VLSM. We subsequently developed and compared radiomics and location features (location omics) models constructed using three machine learning algorithms to identify the model with the optimal prediction performance. Finally, we developed and validated an interpretable ML model to predict END in elderly stroke patients based on the above optimal model. Importantly, we hypothesized that the use of location omics would yield the best accuracy and generalization performance in predicting END by complementarily exploiting location features.

## Methods

### Data acquisition and dataset description

This study included acute stroke patients seen at Chinese hospitals between January 2020 and June 2023. Patients were divided into a training cohort (Nanjing First Hospital) and an independent external validation cohort (The Affiliated Gaochun Hospital of Jiangsu University). This study was approved by the Institutional Ethics Review Boards of the involved hospitals. As this was a retrospective study, the requirement for informed consent was waived.

**Figure 1:**
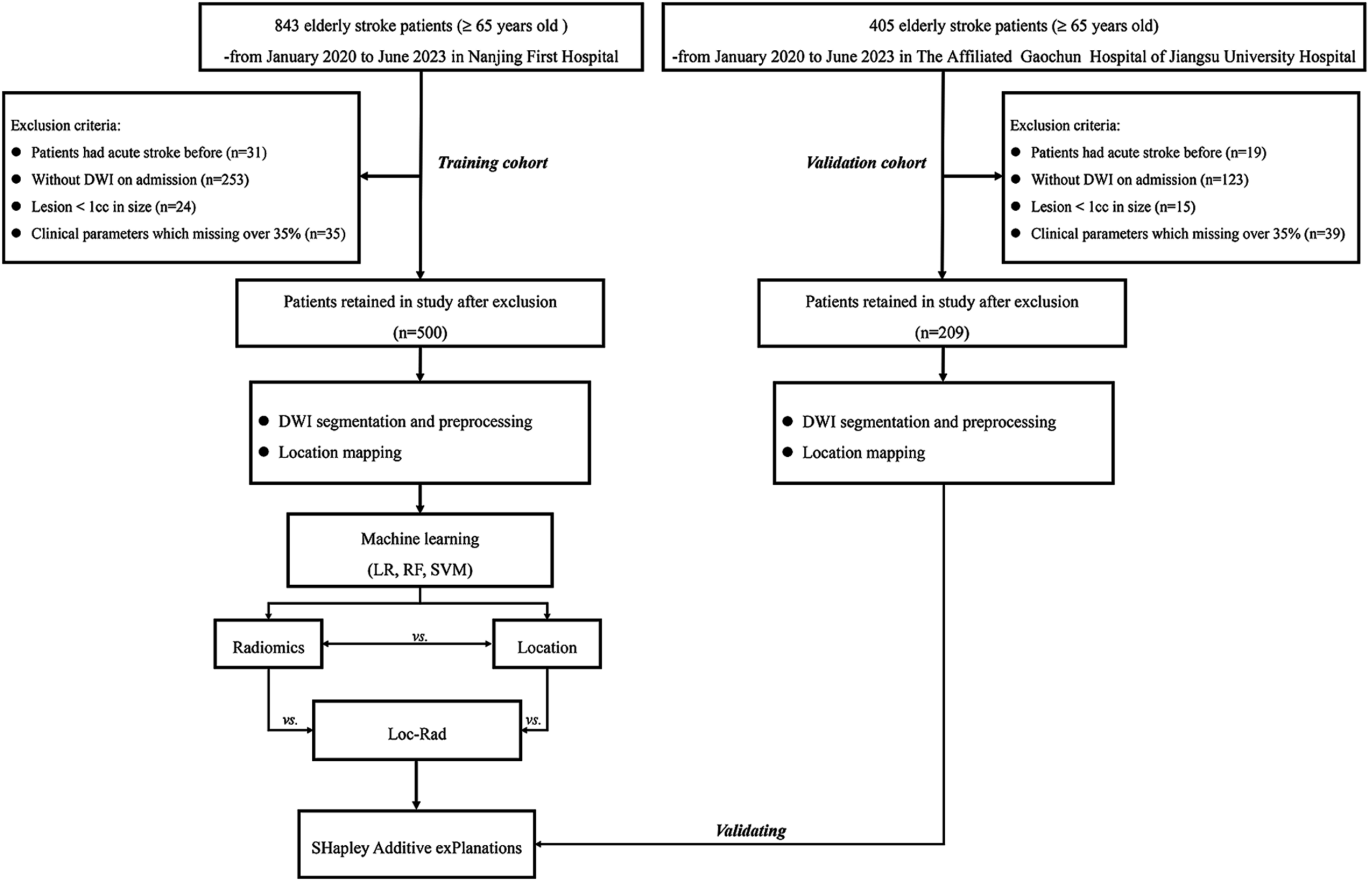
Study flowchart.

Patients were included according to the following criterion: (1) diagnosed as ischemic stroke with 24 h of onset; (2) aged ≥ 65 years old; (3) examined with brain MRI on admission. Patients were excluded if they (1) with excessively small lesions (< 1 cc); (2) MRI with motion artifact. All patients in this study provided written informed consent before MRI examined and received standard stroke treatment [intravenous thrombolysis [alteplase; recombinant tissue plasminogen activator (rt-PA)] (IVT), endovascular treatment (EVT), bridging therapy (both IVT and EVT) or antiplatelet treatment (conservative treatment)]. A detailed flowchart of the patient selection process is provided in Figure 1.

Age, sex, NIHSS score on admission, systolic pressure on admission, diastolic pressure on admission, blood glucose level on admission, HbA1c level, triglyceride level, and any history of hypertension, diabetes mellitus, hyperlipidemia, atrial fibrillation (AF), smoking or drinking were recorded.

END was defined as NIHSS score increase ≥4 NIHSS points within 72 h of symptom onset compared to NIHSS score on admission. The NIHSS score was assessed by a neurologist and was determined by the superior doctor from their assigned medical team. In cases of disagreement in NIHSS scoring, a third neurologist from each center was consulted to reach a final decision.

### Imaging analysis

#### DWI examinations

All admission MR examinations were performed according to standardized protocols. MRI examinations were performed with a 3.0 Tesla MRI scanner (Ingenia, Philips Medical Systems) with an 8-channel receiver array head coil. The MRI protocol included DWI [spin echo (SE) sequence, repetition time (TR), 2,501 ms; echo time (TE), 98 ms; acquisition matrix, 152*122; 3 directions; field of view (FOV), 230 mm*230 mm; flip angle (FA), 90°; slices, 18; section thickness, 6 mm; intersection gap, 1.3 mm; b values, 0 and 1,000 s/mm^2^].

#### DWI segmentation and preprocessing

High-intensity signal infarction areas on DWI images and apparent diffusion coefficient (ADC) < 620 × 10^−3^ mm^2^/s were drawn as volumes of interest (VOIs) using ITK-SNAP.^1^ The infarct volume segmentations were performed together by two neuroradiology staff (CZ, an attending doctor with 5 years of experience in neuroradiology, and MP a director with 15 years of experience in neuroradiology) who were blinded to the clinical data. To facilitate voxel comparison between groups, all images were aligned within the same coordinate space for voxel analysis. The DWI images were normalized to standard MNI coordinates with a cost-function masking approach using SPM 12 software.^2^ All the DWI image registration results were subject to visual quality assessment, and any necessary adjustments were implemented.

#### Location mapping

The frequency of infarct occurrence at each voxel was computed to produce a probabilistic map. To observe the specific patterns of anatomical infarct distribution for different outcomes, probabilistic maps and voxelwise chi-square tests were performed between favorable outcomes and unfavorable outcomes.

### ML model development

#### Location models

The location feature set was constructed by extracting the volumes of infarct lesions for each patient under the Johns Hopkins University (JHU) template, which contains 189 annotated brain regions covering 68 white matter, 110 Gy matter, and 11 ventricle parcellations.^3^ Subsequently, least absolute shrinkage and selection operator (LASSO) to select potential features that were associated with END.

To avoid overfitting, a regularization parameter (*λ*) that controls the strength of the penalty applied to the coefficients were included, and five cross-validation was used to select the optimal value of λ. The individual prediction was conducted by using logistic regression (LR), random forest (RF), and support vector machine (SVM). We used 5-fold cross-validation in the training cohort to choose the optimal parameters and avoid overfitting.

#### Radiomics models

The radiomics features were extracted from the VOIs with PyRadiomics software (version: 3.0.1),^4^ which conforms to the Image Biomarker Standardization Initiative (IBSI). A total of 1,143 features were extracted from the DWI images (shape-based (3D) features, first-order statistical features, GLCM, GLRLM, GLSLM, GLDM). The features selection and prediction models were constructed using the method above.

#### Loc-Rad models

To develop an optimal diagnostic model, we constructed a fusion model by integrating location and radiomics models. We employed average information fusion strategies to amalgamate the prediction scores produced by the location model and the radiomics model, thereby formulating the Loc-Rad model.

In the development of the three models, each incorporated not only the features specific to their respective subsets (radiomics, location features, or integrating location and radiomics features) but also included clinical variables that exhibit statistical differences.

### Statistical analysis

The R software package (version 4.0.3) was used to perform the statistical analyses. The Kolmogorov–Smirnov test was used to assess whether the data followed a normal distribution. Continuous variables are presented as medians (interquartile ranges) and were compared groups with the t test if they were normally distributed or the Mann–Whitney *U* test if not. Categorical variables are presented as percentages and were compared between groups with the chi-square test or Fisher’s exact test. All the statistical tests were two-sided, and *p* values < 0.05 were deemed to indicate statistical significance.

Intra-rater consistency between the two VOI segmentations was calculated using the Dice coefficient. Nine independent ML models (three algorithms, and radiomic, location features) were used to predict END in elderly stroke patients. The area under the curve (AUC) was used to evaluate model performance, and the optimal model was selected for further analysis using Delong’s tests. The performance of the optimal model was evaluated with the sensitivity, specificity, negative predictive value (NPV) and positive predictive value (PPV). The clinical benefit of the models was evaluated with decision curve analysis (DCA). Calibration curves were assessed graphically by plotting the observed rates against the RF-predicted probabilities, and a concordance index (C-index) was calculated via a bootstrap method with 1,000 resamples. Furthermore, we used SHapley Additive exPlanations (SHAP) to interpret and visualize the impact of predictors on END risk based on the best performing model.

## Results

### Patient characteristics

Among the 1,248 patients initially screened, 709 were included for analysis in the present study (500 patients for training set and 209 for validation set). The baseline characteristics of the END group (*n* = 271) and non-END group (*n* = 438) are presented in Table 1. There were statistically significant in age, male, large vessel occlusion (LVO), NIHSS on admission and AF between END and non-END. The Dice coefficient between the two researchers for VOI segmentations reached 0.95.

**Table 1:** Baseline characteristics of the END and non-END groups.

Characteristics	END (*n* = 271)	Non-END (*n* = 438)	*p*-value
Age, median (IQR)	78 (72, 84)	74 (69, 80)	< 0.001
Male, *n* (%)	146 (53.9%)	277 (63.2%)	0.013
LVO, *n* (%)	156 (57.6%)	89 (20.3%)	< 0.001
NIHSS on admission, median (IQR)	12 (6.5, 17)	3 (2, 7)	< 0.001
Hypertension, *n* (%)	215 (79.3%)	341 (77.9%)	0.641
Diabetes, *n* (%)	93 (34.3%)	164 (37.4%)	0.400
Hyperlipemia, *n* (%)	9 (3.3%)	27 (6.2%)	0.094
Smoking, *n* (%)	78 (28.8%)	117 (26.7%)	0.549
Drinking, *n* (%)	51 (18.8%)	85 (19.4%)	0.847
AF, *n* (%)	60 (22.6%)	38 (11.6%)	< 0.001
Therapy, *n* (%)			0.103
EVT	22 (8.1%)	19 (4.3%)	
IVT	76 (28%)	134 (30.6%)	
Conservative treatment	173 (63.8%)	285 (65.1%)	

END, early neurological deterioration; AF, atrial fibrillation; LVO, large vessel occlusion; IVT, intravenous thrombolysis; EVT, endovascular treatment.

### Infarction location probabilistic maps

**Figure 2:**
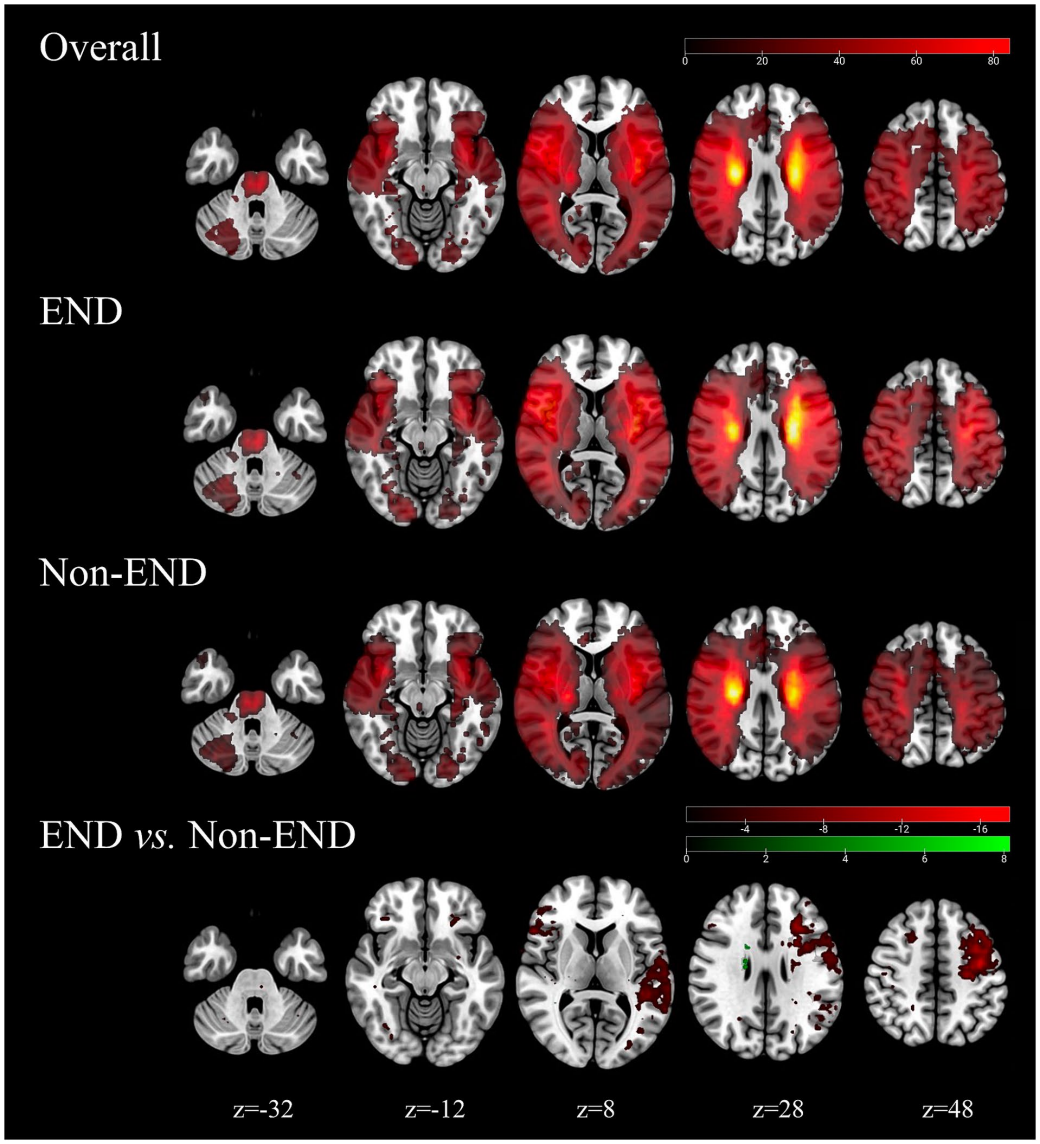
Probability atlas of infarct location in the training cohorts (*N* = 500). The top row depicts the anatomic distribution pattern of all elderly stroke patients. The bottom panels show the location patterns of patients with END (*N* = 438) and with non-END (*N* = 271), and the comparison maps (chi-square test, *p* < 0.05).

An overview of location maps for the END and non-END after stroke are presented in Figure 2. Overall, the infarct lesions were nearly symmetrically, but spatially heterogeneously distributed, with predilection in the blood supply of the bilateral medial lenticulostriate artery, temporal parts of the bilateral MCA, insular portion of the bilateral MCA, occipital pars of the bilateral posterior cerebral artery, the bilateral anterior choroidal and thalamoperforators, and the brainstem of the basilar artery. Infarcts in the left superior frontal gyrus, left middle frontal gyrus, and left central posterior gyrus were associated with END (*p* < 0.05).

### Feature selection

**Figure 3:**
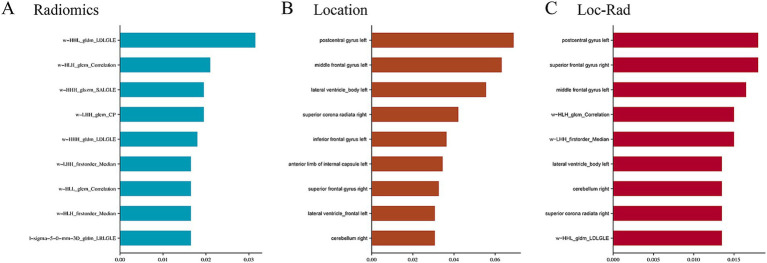
Top nine important features and weights for predicting the END in radiomics **(A)**, location features **(B)**, and Loc-Rad features **(C)**.

A total of 1,143 radiomics features and 189 location features were extracted from the DWI images. The top 9 radiomics features (Figure 3A), location features (Figure 3B), and location-radiomics features (Figure 3C) that were ultimately obtained from the training cohort were screened for further analysis. The best features related to END in the Loc-Rad model were highly consistent with individual radiomics model and location model.

### Performances of models

**Figure 4:**
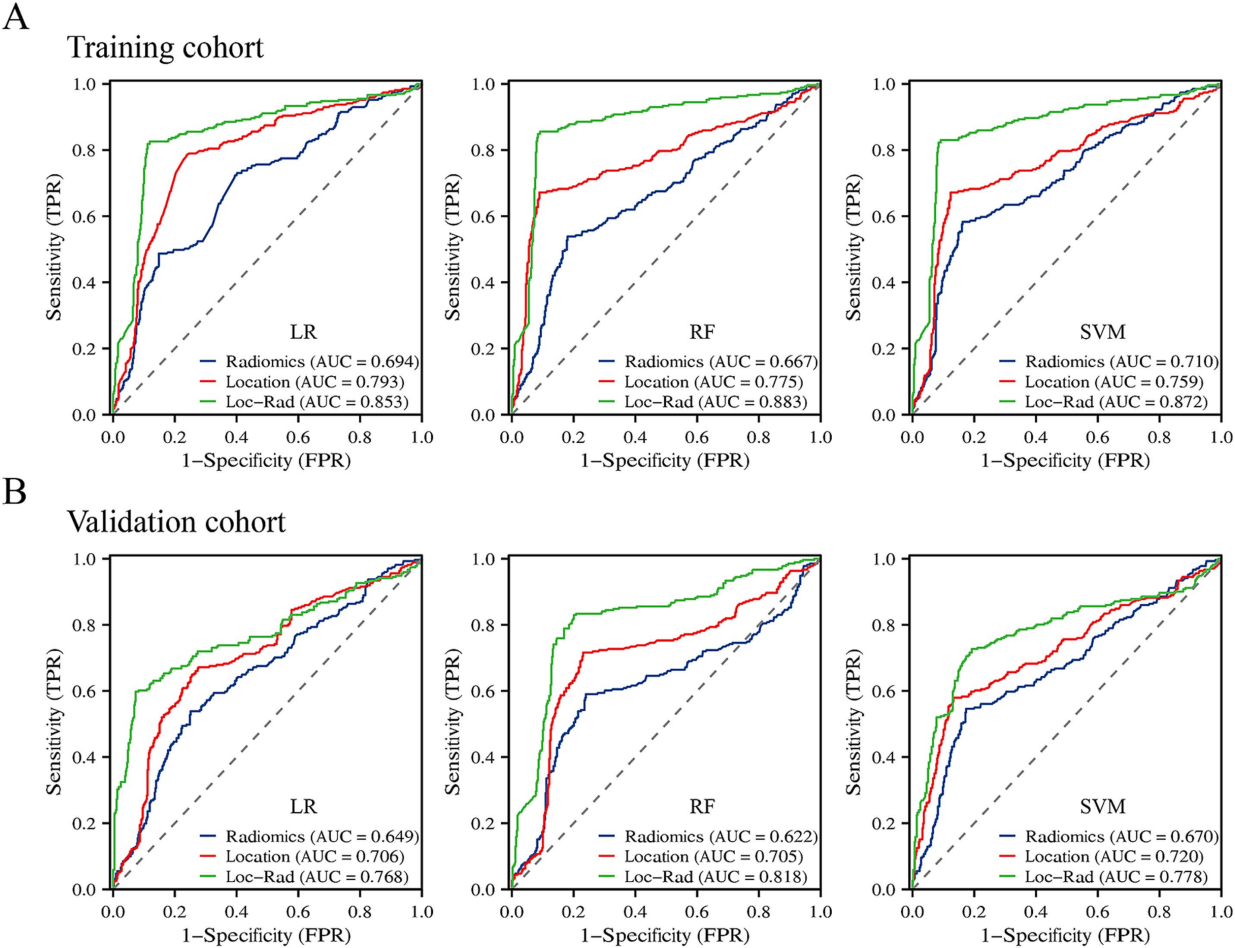
Prediction performance of the three machine learning models for END in the elderly stroke in the training cohort **(A)** and validation cohort **(B)**.

In the training cohort, the 3 machine learning methods used the selected features to train the predictive models. Among the models built from the different feature sets, the Loc-Rad models demonstrated superior performance compared with the location models and radiomics models in the training cohort (Table 2). Delong’s tests demonstrated statistical significance across all tasks (*p* < 0.05). The AUCs of the 3 ML models based on Loc-Rad features ranged from 0.853 to 0.883 in the training cohort (Figure 4), with the RF model yielding the highest AUC [0.883 (95% CI: 0.854–0.912)] (Table 2).

**Table 2:** Performance of different models for predicting END in elderly stroke patients for train cohort.

Classifiers	Features	AUC	Accuracy	Sensitivity	Specificity	PPV	NPV
LR	Radiomics	0.694	0.712	0.487	0.852	0.670	0.729
	Location	0.793	0.770	0.786	0.760	0.670	0.852
	Loc-Rad	0.853	0.860	0.826	0.881	0.811	0.891
RF	Radiomics	0.667	0.712	0.539	0.820	0.649	0.742
	Location	0.775	0.819	0.672	0.911	0.824	0.818
	**Loc-Rad**	**0.883**	**0.888**	**0.856**	**0.909**	**0.853**	**0.911**
SVM	Radiomics	0.710	0.740	0.583	0.838	0.690	0.765
	Location	0.759	0.798	0.672	0.877	0.771	0.812
	Loc-Rad	0.872	0.879	0.830	0.909	0.849	0.896

ML, machine learning; AUC, area under the curve; NPV, negative predictive value; PPV, positive predictive value; LR, logistic regression; RF, random forest; SVM, support vector machine. Best results are marked in bold.

### Independent external validation

**Figure 5:**
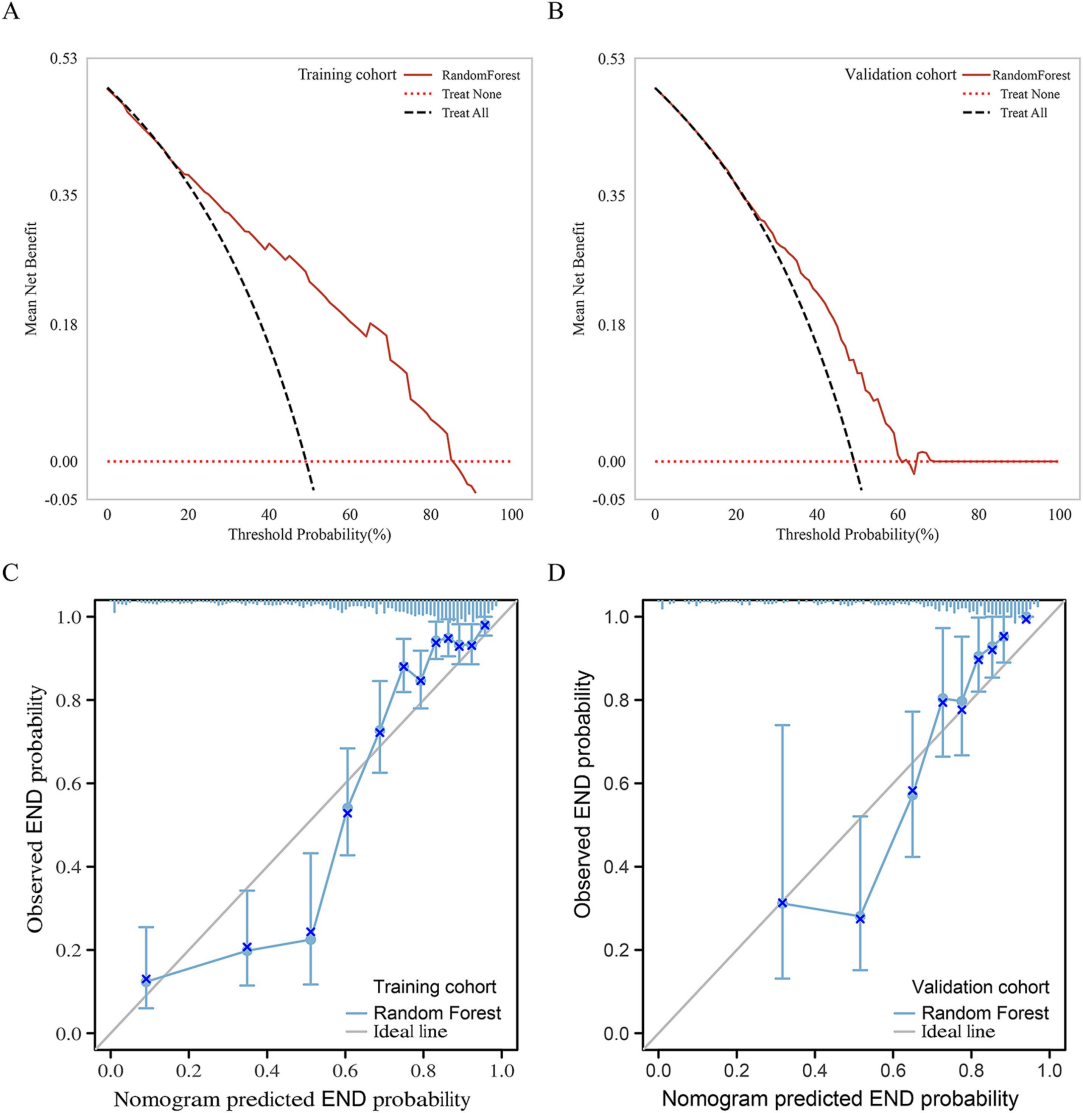
Net benefits of the RF model by decision curve analysis for predicting END in the Training cohort **(A)** and external validation cohort **(B)**. Calibration curves of the nomogram to predict the probability of END in **(C)** the training cohort and **(D)** validation cohort. The actual probability of END is plotted on the y-axis; the RF-predicted probability is plotted on the x-axis.

In the validation cohort, The AUCs of Loc-Rad model in predicting END was 0.768–0.818 (Figure 4). The loc-rad models also were significantly better than that of the location and radiomics models (*p* < 0.05). The RF model yield the highest AUC [0.818 (95% CI: 0.784–0.852)], and the corresponding accuracy, sensitivity, specificity, PPV, and NPV were 0.811, 0.830, 0.799, 0.718, and 0.884 (Table 3). Figure 5 shows the decision curves of Loc-Rad in RF-based model with good performance. The RF model displays a consistent positive net benefit in the training cohort (Figure 5A) and external validation cohort (Figure 5B). The calibration plots for the probability of END were predicted well in both the training cohort (C-index 0.811, 95% CI 0.798–0.825; Figure 5C) and validation cohort (C-index 0.809, 95% CI 0.792–0.828; Figure 5D).

**Table 3:** Performance of different models for predicting END in elderly stroke patients for validation cohort.

Classifiers	Features	AUC	Accuracy	Sensitivity	Specificity	PPV	NPV
LR	Radiomics	0.649	0.669	0.539	0.749	0.570	0.724
	Location	0.706	0.704	0.672	0.724	0.601	0.781
	Loc-Rad	0.768	0.801	0.598	0.927	0.835	0.788
RF	Radiomics	0.622	0.695	0.590	0.760	0.604	0.750
	Location	0.705	0.749	0.716	0.769	0.658	0.814
	**Loc-Rad**	**0.818**	**0.811**	**0.830**	**0.799**	**0.718**	**0.884**
SVM	Radiomics	0.670	0.719	0.546	0.826	0.661	0.746
	Location	0.720	0.757	0.579	0.868	0.730	0.769
	Loc-Rad	0.778	0.777	0.727	0.808	0.701	0.827

ML, machine learning; AUC, area under the curve; NPV, negative predictive value; PPV, positive predictive value; LR, logistic regression; RF, random forest; SVM, support vector machine. Best results are marked in bold.

### SHAP interpretable model

**Figure 6:**
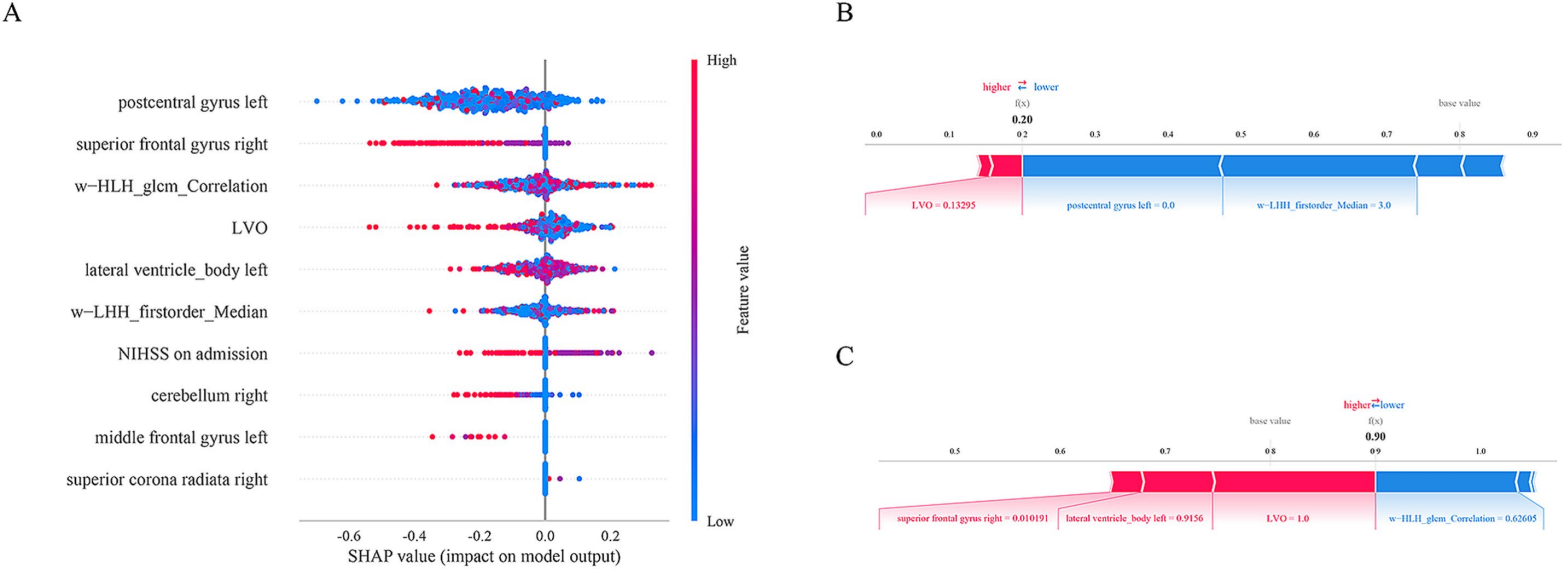
The SHAP plots for the RF model **(A)**. The SHAP plots illustrated that the lower levels of these top 5 predictors (i.e., blue dots) were associated with a lower probability of END (i.e., SHA*p* value<0). Examples of the RF model output for an individual patient with the determining feature values that influenced the classification decision from the non-END **(B)** and the END **(C)**. The contributing variables are arranged in the horizontal line, sorted by the absolute value of their impact. The output value is the predicted risk of unfavorable outcomes. The base value means the expected value of model.

The SHAP method was used to assess the importance of each variable in predicting END of the optimal RF model. The top five variables from the Loc-Rad model in the training cohort included postcentral gyrus left, superior frontal gyrus right, w−HLH_glcm_Correlation, large vessel occlusion and lateral ventricle_body left (Figure 6). Figure 6A shows the SHAP plots for the RF model. The SHAP plots illustrated that the lower levels of these top 5 predictors (i.e., blue dots) were associated with a lower probability of END (i.e., SHAP value<0). Figures 6A,B provides two examples for predicting the risk of END in elderly stroke patients.

## Discussion

In this study, we integrated a coordinatized lesion location analysis into the classical ROI-based analysis of radiomics. By comparing with single models of location and radiomics, we found that the fusion Loc-Rad model combining location and radiomic features demonstrated a high AUC (0.883) and accuracy (0.888) when constructed with the RF algorithm; a similar prediction performance was achieved in the validation cohort (AUC: 0.818; accuracy: 0.811), indicating the high clinical utility and generalizability of the prediction model. The results of this study also indicated that the postcentral gyrus left, superior frontal gyrus right, w−HLH_glcm_Correlation, large vessel occlusion and lateral ventricle_body left were important contributing factors to model performance. These findings contribute to the early identification of elderly stroke patients at high risk of END and who should receive targeted clinical care through timely interventions.

The correlation between brain lesion location and functional outcomes has been extensively studied (Biesbroek et al., 2017), with early case series from the 19th century documenting individuals with a specific deficit who had a lesion at a particular location. To date, more than 100 studies have applied modern lesion-symptom mapping techniques to analyze associations between infarct location and cognition at the group level (Biesbroek et al., 2017; Ding et al., 2023). In contrast, few studies have assessed the predictive value of infarct location for END in elderly patients. In our study, we enrolled 709 elderly stroke patients with lesions that covered the vast majority of the volume of the brain, allowing us to evaluate and externally validate the predictive value of most infarct locations across the brain. We found that the left superior frontal gyrus, left middle frontal gyrus, and left central posterior gyrus were associated with unfavorable outcomes. The left central posterior gyrus played a vital role in integrating sensory inputs, which are essential for motor planning, coordination, and the perception of body position and movement (proprioception). Damage to the central posterior gyrus can lead to somatosensory deficits, such as impaired tactile sensation, proprioception, or the ability to perceive pain and temperature. Studies have shown that the extent of sensory impairment following a stroke is a strong predictor of motor recovery (Kessner et al., 2019). Patients with preserved somatosensory function tend to have better motor outcomes, as sensory feedback is essential for relearning motor skills during rehabilitation. Conversely, severe sensory deficits can limit the effectiveness of motor rehabilitation, as the brain struggles to integrate sensory information necessary for movement (Lamorie-Foote et al., 2024).

Previous studies have shown that malnutrition, Cystatin C, internal carotid artery occlusion, and brain atrophy can be used to assess the END of stroke patients (Bao et al., 2022; Kim et al., 2017; Boulenoir et al., 2021; Tschirret et al., 2018). However, most existing studies have not reported the prognostic value of these factors, making it difficult to identify elderly stroke patients with an END risk in the early stages in terms of accurate evaluation. Advances in ML technology have allowed clinicians to utilize large datasets to develop effective models for improving disease identification (Heo et al., 2019; Sung et al., 2020; Kim et al., 2021; Sheth et al., 2023). An increasing body of research has focused on the development of ML models for evaluating functional outcomes in stroke patients (Sung et al., 2020; Kim et al., 2021). While current predictive models for early neurological deterioration (END) in stroke patients often rely on clinical factors or imaging biomarkers alone, which may not fully capture the complex interplay between lesion location and radiomic features, leading to moderate predictive accuracy. We applied classifiers constructed from three algorithms to predict END in elderly stroke patients. We found that the ML models achieved an AUC of 0.667–0.710 when constructed with radiomics. However, we observed that adding location features significantly increased the AUC to 0.853–0.883. And the fusion Loc-Rad model combining location and radiomic features demonstrated a high AUC (0.883) and accuracy (0.888) when constructed with the RF algorithm. The RF algorithm’s ability to handle complex, non-linear relationships, its robustness to overfitting, its natural feature importance ranking, and its ability to handle imbalanced data make it a superior choice for predicting END in elderly stroke patients compared to LR and SVM. Wu et al. (2015) reported that for a given age and sex, the risk that a patient would have greater long-term disability depended on the location of the infarct. This effect appears to have a lesser impact on the admission NIHSS score, indicating that lesion location may play a distinct role in determining stroke severity independent of other factors. Our study addresses these limitations by integrating both location and radiomic features into a ML model, significantly enhancing predictive performance and providing a more comprehensive tool for identifying high-risk elderly stroke patients.

Our study utilized the SHAP method to enhance the interpretability of the machine learning models at the cohort and individual patient levels, employing user-friendly visualization tools for demonstration purposes. We found that the postcentral gyrus left, superior frontal gyrus right, w−HLH_glcm_Correlation, large vessel occlusion and lateral ventricle_body left were the five most important factors in the RF model, and these features related to END in the Loc-Rad model were highly consistent with individual radiomics model and location model. The SHAP value for a feature was calculated as a weighted average of the differences in predictions when the feature was included versus excluded from all possible subsets. The weights are determined by the number of ways a subset of a certain size can be formed. SHAP values provided a measure of the importance of each feature for a specific prediction. A higher absolute SHAP value indicated that the feature has a greater impact on the prediction. Through the use of SHAP, the interpretable machine learning model developed in this study addresses the issue of trust between clinical doctors and artificial intelligence algorithms and helping identify END in elderly stroke patients so that they can be directed toward intervention therapies early in their treatment course.

Although our study yielded promising results, it is crucial to acknowledge its limitations. The proportion of END in elderly stroke patients was relatively low, resulting in an imbalance between the two groups. Further augmentation of the sample size of patients with END is necessary to validate our findings. Second, this is a retrospective study, it is susceptible to selection and recall bias. Future studies should aim to address these biases by employing prospective designs, ensuring more comprehensive data collection, and validating the models in diverse populations. In addition, although external validation was used in this study, we did not perform the models on data from a different time period, which would better demonstrate the model’s robustness and applicability over time. Finally, the Loc-Rad and ML models were constructed using data mainly from Chinese patients, and therefore validation across various racial groups is warranted to ascertain their applicability and generalizability. Prospective international multicenter studies are necessary for additional validation of the performance of the RF model.

## Conclusion

In conclusion, our study provides the first comprehensive maps of infarct location, associated with a high risk of END in elderly stroke patients. Location features were generated from the maps and subsequently integrated into an ML model. The RF model based on location features and radiomics constructed in our study achieved stable prediction performance across different cohorts. Owing to its high accuracy and reliability, the RF model based on location features and radiomics could be applied by clinicians to identify individual patients at risk of END after stroke in elderly patients.

## Data Availability

The raw data supporting the conclusions of this article will be made available by the authors, without undue reservation.

## Footnotes

^1^ www.itksnap.org

^2^ http://www.fl.ion.ucl.ac.uk/spm/

^3^ https://github.com/JLhos-fmri/ClinicalFrequencyAnalysisToolkit

^4^ https://pyradiomics.readthedocs.io/en/latest/
